# Improving TB case notification and treatment coverage through data use

**DOI:** 10.5588/pha.22.0001

**Published:** 2022-09-21

**Authors:** O. O. Chijioke-Akaniro, E. Ubochioma, A. Omoniyi, O. Fashade, O. Olarewaju, S. Asuke, E. C. Aniwada, A. N. Uwaezuoke, J. Sseskitooleko, N. Workneh, E. Masini, B. Morris, A. Lawanson, C. Anyaike

**Affiliations:** 1 National Tuberculosis, Leprosy and Buruli Ulcer Control Programme, Abuja, Nigeria; 2 World Health Organization Country Office, Nigeria; 3 Bingham University, New Karu, Nigeria; 4 University of Nigeria Teaching Hospital, Ituku Ozalla, Enugu, Nigeria; 5 The Global Funds to Fight AIDS, Tuberculosis and Malaria, Geneva, Switzerland

**Keywords:** secondary data, tuberculosis services, programme

## Abstract

**BACKGROUND:**

This was a study on national TB data.

**OBJECTIVE:**

To assess improvement in TB case notification and treatment coverage through improved data use for action in Nigeria

**DESIGN:**

We analysed pre- and post-intervention secondary TB programme data comprising data on increased supervisory visits, incentives for health workers, DOTS expansion, outreaches and geo-code monitoring. Trend analysis was performed using Cochran-Armitage χ^2^ test for linear trends.

**RESULTS:**

Case-finding increased from 104,904 cases in 2017 to 138,591 in 2020. There was an increase of 2.0% from 2017 to 2018, 13.0% in 2018 to 2019 and 15.0% in 2019 to 2020 (*P* < 0.001). Facility DOTS coverage increased from 7,389 facilities in 2017 to 17,699 in 2020. There was an increase of 30.0% in 2018, 31.0% in 2019 and 40.0% in 2020 (*P* < 0.001). The number of reporting facilities increased from 5,854 in 2017 to 12,775 in 2020. Compared with 2017, there were an increase of 20.0% in 2018, 38.0% in 2019 and 32.0% in 2020 (*P* < 0.001). Treatment coverage rate increased from 24% in 2018 to 27% in 2019 and 30% in 2020.

**CONCLUSION:**

TB service expansion, improved monitoring and the use of data for decision making are key in increasing TB treatment coverage.

## Abstract (fr)

**CONTEXTE:**

Il s’agit d’une étude sur les données nationales relatives à la TB.

**OBJECTIF:**

Évaluer l’amélioration de la notification des cas de TB et de la couverture du traitement grâce à une meilleure utilisation des données pour l’action au Nigéria.

**MÉTHODE:**

Nous avons analysé les données du programme secondaire de lutte contre la TB avant et après l’intervention, y compris les données sur l’augmentation des visites de supervision, les mesures incitatives pour les travailleurs de la santé, l’expansion du système DOTS, les activités de proximité et la surveillance des codes géographiques. L’analyse des tendances a été réalisée à l’aide du test du χ^2^ de Cochran-Armitage pour les tendances linéaires.

**RÉSULTATS:**

La recherche de cas est passée de 104 904 cas en 2017 à 138 591 en 2020. On observe une augmentation de 2,0% de 2017 à 2018, de 13,0% de 2018 à 2019 et de 15,0% de 2019 à 2020 (*P* < 0,001). La couverture DOTS des établissements est passée de 7 389 établissements en 2017 à 17 699 en 2020. On observe une augmentation de 30,0% en 2018, 31,0% en 2019 et 40,0% en 2020 (*P* < 0,001). Le nombre d’installations déclarantes est passé de 5 854 en 2017 à 12 775 en 2020. Par rapport à 2017, il y a eu une augmentation de 20,0% en 2018, 38,0% en 2019 et 32,0% en 2020 (*P* < 0,001). Le taux de couverture du traitement est passé de 24% en 2018 à 27% en 2019 et 30% en 2020.

**CONCLUSION:**

L’expansion des services de lutte contre la TB, l’amélioration de la surveillance et l’utilisation des données pour la prise de décision sont essentielles pour augmenter la couverture du traitement de la TB.

TB is a global public health problem of major concern. It is among the leading infectious causes of death, and one of the most important socio-economic diseases worldwide.^1^ It was declared a global public health emergency in 1993.^2^ Since then, commitments with actionable targets to end TB epidemic have been operationalised; however, the pace of progress in most regions and countries is insufficient.^3^ For example, DOTS regimens were introduced and implemented in 182 countries, which led to the enrolment of more than 20 million patients on treatment and cure of more than 16 million of them in 2004.^2^ The Stop TB Partnership aims to eliminate TB by 2050, and Sustainable Development Goals 3 specifically has a target to end the TB epidemic in all countries by 2030.^4^

Despite this, in 2020 approximately 9.9 million people fell ill with TB and an estimated 1.3 million died globally.^4^ In the same year, Africa accounted for 25% of TB patients worldwide.^4^ The global TB treatment success rate was 86% among all new TB cases.^5^,^6^ Nigeria is ranked sixth globally, first in Africa in terms of TB burden among the eight countries accounting for two-thirds of the global total TB cases; it also figures in the list of 30 countries with the triple high burden of TB, TB-HIV and multidrug-resistant TB.^7^–^9^ In 2018, about 452,000 people in Nigeria had TB, with an estimated incidence rate of 219/100,000 and mortality rate of 62/100,000, excluding those who are HIV-positive.^10^,^11^ Drug-resistant TB (DR-TB) is estimated at 21,000 incident cases, of which 2,306 were detected and notified in 2018. Likewise TB-HIV co-infection incidence rate was 27/100,000 and prevalence was 19.1%.^5^,^11^

The Nigerian Tuberculosis, Leprosy and Buruli Ulcer Control Programme (NTBLCP) coordinates TB, Leprosy and Buruli ulcer. It was established to reduce the burden, socio-economic impact and transmission of these diseases in Nigeria.^12^ The number of health facilities providing DOTS services as of 2004 (year of commencement) was 5,389; this rose to 7,389 in 2017.^13^ These successes notwithstanding, the major problem with TB in Nigeria is poor case-finding, and the consequent poor treatment coverage rate.^14^ For example, in 2017, only 104,904 TB cases were notified out of 407,000 expected in that year. This translates to a treatment coverage rate of 25.8%, leaving a gap of 302,096 patients who were either undetected or detected but not notified.^14^ Low TB service coverage has been identified as the major factor in the underdiagnosis of TB in Nigeria.^15^

Moreover, the country’s treatment coverage rate has remained stagnant at about 24% since 2016, with over 300,000 missed TB cases.^16^ Increasing TB service delivery points and finding the missing TB cases in order to change the static treatment coverage was therefore a priority for the NTBLCP. In 2017, approximately 6.4 million TB cases were newly diagnosed and notified globally, Nigeria was second among five countries that are responsible for half of the global gap, accounting for 11% of this gap.^17^ A study in Lagos State, Nigeria, in 2017 revealed that a minimum of 60% of all health facilities underreported TB, with an average of 7.4% underreporting between health facility records and that of the National TB Programme (NTP).^18^ This is likely the situation in other states of the Federation.

These data informed the intervention strategies employed by NTBLCP on non-reporting facilities with the aim of improving case-finding, case notification and the treatment coverage rate, as well as the number of facilities reporting TB activities.

## METHOD

This study covered all states of the country and the Federal Capital Territory. This was a secondary data analysis of national data collected by the NTBLCP programme before and after a series of interventions used to improve the NTP. During first two quarters of 2017, national data were analysed to identify none or poorly performing facilities. The reasons for these as perceived by health workers were documented. These included lack of knowledge about TB at the community-level, patient attrition, poor coverage of TB services and attitude of health workers. During the remaining quarters of the same year, the reasons identified were addressed using diverse methods, with emphasis on changing strategies and standard of operations or improvement on promising existing ones.

Consequently, NTBLCP liaised with the State Programme Managers (SPMs), as well as with Local Government TB supervisors (TBLS) to reach out to the non-reporting facilities identified using a pre-developed, one-page standard operation procedure (SOP). There was improved supervisory visits by TBLS and SPMs to ensure adequate monitoring and supervision of health workers at facilities. Selection of sites for supervision was such that 60% of sites per state were non-reporting facilities. Six states were supervised every quarter at the national or central level (NTP), for a total of 24 states each year; eight facilities from each state were supervised, for a total of 192 facilities. Supervisors were expected to visit all the facilities in each local government area (LGA) and incentivise non-reporting ones.

Incentives (transport allowance and stipends) were provided to all 774 LGA supervisors for visiting facilities and health workers. The supervision stipends were paid in full for reporting facilities, but only 50% (twice) for non-reporting facilities. LGA supervisors are responsible for ensuring that facilities report to increase the total funds they received at the end of each quarter.

Outreaches were organised at community level by TB focal persons and health workers to increase awareness and combat patient attrition. Areas with increased numbers of identified cases serve as outreach hotspots. Health facilities are funded on a monthly or a quarterly basis depending on identified need.

Geo-code monitoring using Open Data Kit tool was implemented to ensure availability of real time information on the visits to health facilities. These codes serve as evidence and are used to guide for payment of incentives. DOTS site coverage was increased to approximately 40%, including private health facilities.

Data were extracted using an MS Excel proforma (MicroSoft, Redmond, WA, USA) from the NTBLCP database. This was exported, edited and analysed using Statistical Package for Social Sciences (SPSS) v25 (IBM Corp, Armonk, NY, USA). Data were complete, as these were extracted from the national database, with quality-assured data collection and reporting pathway. The absence of data at the facility level was not deemed substantial enough to affect the results. Changes over 2018 to 2020 were compared with 2017. Trend analysis was performed using Cochran-Armitage χ^2^ test for linear trends significance level of *P* < 0.05. Tables and charts were used in presenting the data

Ethical clearance and informed consent were not required as the study was a de-identified data evaluation of pooled records of patients from the national database, with no direct contact with clients. However, confidentiality and security of the data were ensured.

## RESULTS

**FIGURE 1.**
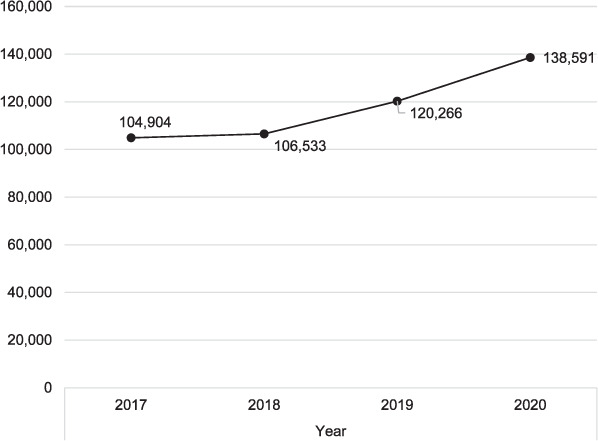
TB case-finding, 2017–2020.

**FIGURE 2.**
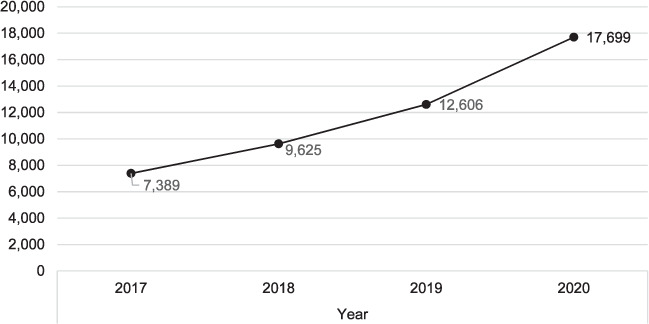
TB treatment coverage, 2017–2020.

**FIGURE 3.**
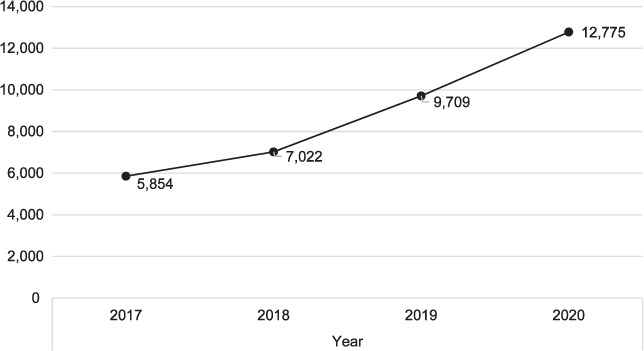
TB reporting facilities, 2017–2020.

The Table shows that case-finding increased from 104,904 cases in 2017 to 138,591 in 2020 (Figure 1). This increase was minimal from 2017 to 2018 but rapid from then to 2020. There was an increase of 2.0% from 2017 to 2018, 13.0% from 2018 to 2019 and 15.0% from 2019 to 2020 (*P* < 0.001). DOTS facilities coverage increased from 7,389 cases in 2017 to 17,699 in 2020 (Figure 2). There was an increase of 30.0% in 2017 to 2018, 31.0% in 2018 to 2019 and 40.0% in 2019 to 2020 (*P* < 0.001). The number of reporting facilities increased from 5,854 cases in 2017 to 12,775 in 2020 (Figure 3). There was an increase of 20.0% in 2017 to 2018, 38.0% in 2018 to 2019 and 32.0% in 2019 to 2020 in the number of health facilities (*P* < 0.001). Treatment coverage rate increased from 24% in 2018 to 27% in 2019 and 30% in 2020 (*P* < 0.001).

**TABLE:** Distribution of TB parameter performance, 2017–2020 *

Variables	2017 (Baseline)^†^	2018	2019	2020	Total (2018–2020)
Case-finding	104,904	106,533	120,266	138,591	365,390
Percentage of total, %		29.2	32.9	37.9	100.0
Percentage increase, %		2.0	13.0	15.0	
			χ^2^ = 6,328.47, *P* < 0.001		
Treatment (DOTS facilities) coverage	7,389	9,625	12,606	17,699	39,930
Percentage of total, %		24.1	31.6	44.3	100.0
Percentage increase, %		30.0	31.0	40.0	
			χ^2^ = 3,673.34, *P* < 0.001		
Case notification	53,291	53,065	56,690	60,495	170,250
Percentage of total, %		31.2	33.3	35.5	100.0
Percentage increase, %		–0.4	7.0	7.0	
			χ^2^ = 729.58, *P* < 0.001		
Reporting facilities	5,854	7,022	9,709	12,775	29,506
Percentage of total, %		23.8	32.9	43.3	100.0
Percentage increase, %		20.0	38.0	32.0	
			χ^2^ = 2,523.83, *P* < 0.001		

*Percentage increase is the approximate percentage change from the previous year; total is the aggregate from year 2018 to 2020.

^†^Year 2017 was the reference or baseline year before intervention.

## DISCUSSION

In addition to a host of activities undertaken by NTBLCP, TB control requires local solutions that are driven by evidence and local leaders, informed by the needs of at-risk populations and capacities of the local health systems, and supported by the engagement of all relevant stakeholders. This was the reason for developing the NTBLCP interventions.

Our study results show that TB case-finding significantly increased over the years. This is likely due to the quality monitoring interventions deployed by NTBLCP, which is commendable. This implies that if the interventions are sustained and further scaled up, it is possible to demonstrate greater success on TB case notification in Nigeria and closing the gap on missing cases. These successes notwithstanding, documented evidence has shown that the main problem with TB in Nigeria is the low rate of diagnosis and lack of adequate knowledge about TB.^14^ Post-intervention, only 27% and 30%, respectively, of the estimated number of individuals infected with TB who developed TB disease were detected in 2019 and 2020. Similarly, of the estimated drug-resistant TB cases in Nigeria, approximately 10% were put on treatment.^15^

These undetected cases pose a great threat, as they serve as a reservoir for the continued transmission of TB. In a year, a person living with TB disease can infect up to 10 to 15 close contacts.^19^ The NTBLCP in 2019 thus declared the identification of undiagnosed TB patients to be a priority.^15^ The Centers for Disease Control and Prevention (CDC) and the global community working to end TB have also made the finding of these missing cases and interrupting the cycle of transmission their top priority.^19^ According to the National Strategic Plan for TB Control in Nigeria, 2015–2020, the biggest challenge facing TB control in Nigeria is the lack of access to TB diagnosis and treatment for all Nigerians.^13^ In summarising these findings, the WHO concluded that the five countries, including Nigeria, that account for half of the global gap should accelerate efforts to reduce underreporting and enhance access to diagnosis and treatment.^2^,^17^

However, the CDC stated that finding these missing cases and breaking the cycle of transmission requires a strong healthcare system, a public health workforce that can reach those who need care, the laboratory capacity to quickly and effectively diagnose the disease, innovative approaches to meet people at places of care and the expansion of access to TB diagnostic and treatment services.^19^ This gives further credence to outreaches organised in non-performing communities, with the aim to increase awareness and combat patient attrition, which have been found to be a major factor for low case-finding.

Based on the study findings, the number of TB service delivery points (normally referred to as DOTS sites) increased during the intervention years in Nigeria. Poor coverage has been linked to low awareness, as shown in Nigeria where limited health facilities are involved in the delivery of TB treatment services.^15^ One of NTBLCP’s TB control strategies was to find more people with TB by strengthening existing facilities, extending TB care to non-participants, and involving private health facilities. This led to an increase in number of health facilities offering TB treatment services. Majority of the facilities (72%) involved in the exercise are public health facilities. Nevertheless, a strategic approach should be used in incorporating the growing private and informal health sectors in TB services in many countries. These sectors often do not have access to or use quality-assured diagnostics or the anti-TB drugs needed to appropriately diagnose and cure patients, which can lead to underdiagnosis or inappropriate treatment, and ultimately, contribute to drug resistance.^19^ Hence, engaging this sector requires instituting both quality diagnostics and treatment.

This increase notwithstanding, report has shown that less than a third (31%) of health facilities in Nigeria provide TB treatment services as of 2019.^15^ The report further stated that this low coverage of TB treatment services is a key factor in the underdiagnosis of TB in Nigeria.^15^ The Stop TB Strategy has therefore advised Nigeria on the need to improve DOTS coverage in health facilities.^18^ The National TB Prevalence Survey also documented that, despite the implementation of DOTS for many years in Nigeria, services have not penetrated the communities; the survey recommended that NTBLCP should thus consider decentralising TB care and control services to the community.^20^ NTBLCP strove to achieve this with geo-code monitoring, which was implemented to ensure availability of real time information on the visits to health facilities and the expansion of DOTS sites (now covering 44% of the total number of health facilities in the country).

Case notification as well as the number of reporting facilities significantly increased. There was a decline of 4% from 2017 to 2018 and an increase of 7.0% in both 2019 and 2020 in case notifications. Ensuring adequate monitoring and supervision of health workers at facilities helped in bridging the gap. The observed increase in TB case notifications notwithstanding, a large gap still exists between the number of people newly diagnosed and those reported. This gap has been attributed to the underreporting of people diagnosed with TB and underdiagnosis. Underreporting impedes proper understanding of the disease burden and its impact on the response and control interventions.^21^,^22^

TB underreporting is a global problem, with 40% of the cases worldwide never being reported to the NTPs or the public health system.^23^ This is disturbing, as complete reporting and quality surveillance systems for TB are central to the planning, implementation and evaluation of the control strategies, and for determining the real burden of TB.^24^ Mandatory TB notification is one of the integral elements of the overall regulatory framework essential for the implementation of the End TB Strategy. This is essential for routine surveillance and for verifying the TB burden in a community or country.^22^,^25^

A limitation of the study is that results were blanket national findings. Disaggregation at state level would have added to the quality of the study. However, the study was designed to be national. We recommend that a study be designed and conducted to cover this gap.

## CONCLUSION

Interventions employed increased case-finding, DOTS coverage and the number of reporting facilities in recent years. Expansion of facilities providing TB services and improved monitoring and the use of data for decision-making are major factors that contributed to this increase in TB treatment coverage. This should be maintained and improved upon in order to eliminate TB.
